# The Involvement in Domestic Violence and the Severity of Legal, Moral and Social Consequences for the Perpetrators in the Perceptions of Students in Poland and Belarus

**DOI:** 10.3390/ijerph20064947

**Published:** 2023-03-11

**Authors:** Marta Giezek, Andrei Shpakou, Paulina Zabielska, Beata Karakiewicz

**Affiliations:** 1 Subdepartment of Social Medicine and Public Health, Department of Social Medicine, Pomeranian Medical University in Szczecin, 71-210 Szczecin, Poland; 2 Department of Theory of Physical Culture and Sports Medicine, Faculty of Physical Education, Yanka Kupala State University of Grodno, 230023 Grodno, Belarus; 3 Department of Integrated Medical Care, Faculty of Health Sciences, Medical University of Bialystok, 15-089 Bialystok, Poland

**Keywords:** violence, perpetrator of violence, victim of violence, Europe, students

## Abstract

Domestic violence is sequential, developmental and dynamic. The aim of this study was to examine whether, in the perceptions of students in Poland and Belarus, there is a relationship between involvement in violence and the legal and social consequences for the perpetrators. A total of 482 university students took part in the study, including 251 students from Poland and 231 students from Belarus. Statistically, Polish respondents were more frequently involved in domestic violence as witnesses and victims, which was confirmed by χ^2^ test. Based on the 95% confidence interval (CI), it can be concluded that the largest number of respondents from both countries surveyed who have been involved in violence as witnesses (85.2–94.8) indicated that an adequate punishment for perpetrators of violence is imprisonment. Students who have never been involved in domestic violence indicated social consequences as appropriate punishment for the use of violence more often than those who have been involved in violence as witnesses, victims or perpetrators. Witnesses and victims were not found to be in favour of more severe punishment or more serious moral and social consequences than perpetrators. The largest number of respondents indicated that the appropriate consequence of using violence should be imprisonment, followed by a restraining order and eviction from the place of residence.

## 1. Introduction

Domestic violence can be described as sequential, developmental and dynamic. The involvement in domestic violence can occur in three forms: as a victim—a person experiencing violence, as a perpetrator—a person using violence, or as a witness. The effects of domestic violence are also multi-faceted, and are different for victims, for perpetrators and for witnesses [1]. However, each form of involvement in violence is characterised by four perspectives:

Legal perspective—according to the law, violence is a crime and those who commit acts of violence may be punished with imprisonment [2].

Moral perspective—violence means hurting the weaker and is a moral evil. The use of violence should result in sanctions of one’s own conscience and in condemnation by society [3]. A critical moral assessment of violence should give rise to change: in the case of perpetrators, to stop using violence, and in the case of victims, to seek assistance which witnesses should feel willing to provide.

Psychological perspective—reveals the mechanisms responsible for violence [4], and shows the complexity of interactions between perpetrators and victims [5].

Social perspective—an analysis of factors contained in attitudes and customs which may promote or justify violence [3,4,5].

In the studies of domestic violence one fact prevails: that the source of danger is a close person. Regardless of the form of harm suffered, this situation results in many different consequences for victims such as emotional, somatic, cognitive, behavioural and interpersonal consequences, and the effects of violence can persist even after its cessation. Equally complex are problems experienced by perpetrators of domestic violence. Studies of this topic indicate many factors which contribute to the use of violence as a way of functioning in the family [6,7]. The perpetrators have and use an advantage (physical, mental or economic) over victims, which leads to the violation of victims’ fundamental rights and freedoms, causes suffering and puts them at risk of losing their health or lives [8]. It is a public responsibility to respond to the harm of people experiencing violence, which is an offence prosecuted ex officio. In the Polish legal system, this social obligation (without punitive sanctions) is imposed by Art. 304 § 1 of the Code of Criminal Procedure: “Whoever learns that an offence prosecuted ex officio has been committed, shall be under civic duty to inform the state prosecutor or the police” [9]. 

The selection of the countries and respondents for this study is intentional and justified. Despite the fact that both countries have Slavic roots, they have different political systems—democratic in Poland, and authoritarian in Belarus. In Poland, there is the Act of 29 July 2005 on the prevention of domestic violence, as well as the procedure introduced by the Regulation of the Council of Ministers of 13 September 2011 on the “Blue Cards” procedure and the “Blue Card” sample forms. In Belarus, the legislative bills on combating domestic violence drafted by the Ministry of Interior in 2002 and then in 2018 were not passed—they were defeated in public debate. In both countries, domestic violence is considered a crime. The results provided by the respondents of the countries under study might have been affected not only by the differences in political systems between Poland and Belarus, but also by the public authorities’ attitude to the problem of domestic violence. The president of Belarus criticized the Act on counteracting domestic violence, and called it “idiocy of mainly Western origin” in an official interview [10]. At the same time, Poland adopted further amendments to the Act on counteracting domestic violence which introduced separate, quick proceedings in order to force the perpetrator to leave the home jointly occupied with the victim and to ban the perpetrator from approaching the home and its surroundings. The order to leave the home will be enforced—including the possibility of use of coercive measures—regardless of the perpetrators’ possible claims that they have no place to move to. In view of such large differences between Poland and Belarus, and on the other hand similarities—as both countries consider violence a crime—it seems justified to examine the approach of young people to the subject of research. 

The respondents are students of medical departments at the universities in Szczecin (PL) and Grodno (BY) who, as future healthcare workers, will be an important link in domestic violence prevention. 

The aim of the study was to examine whether, in the perceptions of students in Poland and Belarus, there is a relationship between the involvement in violence and the severity of legal and social consequences for the perpetrators. 

## 2. Materials and Methods

A total of 482 university students took part in the study, including: 251 students (196 female and 55 male) of the Department of Health Sciences of the Pomeranian Medical University in Szczecin, Poland, and 231 students (155 female and 76 male) of the Yanka Kupala State University of Grodno, Belarus. The method was a diagnostic survey using the authors’ original questionnaire. The questionnaire consisted of two parts: the first, containing 6 demographic questions, and the second, containing 25 mainly closed-ended questions. The questionnaire for students from Grodno was translated into Belarusian by means of back translation. The tool was provided for students to complete in an online version.

The calculations were made using the R statistical calculation environment (R-3.6.1 for Windows) and a Microsoft Excel spreadsheet, using the standard functions of this program. Due to the fact that the distribution was different than normal (distributions of variables were tested with the Shapiro–Wilk test), descriptive statistics included the characteristics of variables using the mean (M), standard deviation, median (Me), maximum and minimum (Max.–Min.) values and percentile values (25–75% quartiles). The nonparametric Mann–Whitney U test for medians was used to compare differences between the groups. The distribution of qualitative variables was shown by means of frequency and proportion with 95% confidence intervals. Statistical comparisons were completed using the Chi-square test of independence. Significant differences were defined as *p* < 0.05. 

## 3. Results

The participation in the study was voluntary and anonymous. Students were provided with a link to the survey questionnaire, which they could complete in convenient conditions regarding the place and time. It was considered that this form of data collection, bearing in mind the very personal nature of the research, would be the most comfortable for the respondents. In the phenomenon of violence, the roles of “perpetrator” and “victim” show exactly who does what: perpetrator causes violence, and the victim suffers the harm; the perpetrator attacks and the victim is in defence [11]. Violence is accompanied by many strong emotions. People experiencing violence feel fear, shame, humiliation; they are often financially dependent on the perpetrator, have lost hope of receiving assistance, or do not want to break religious and cultural traditions. The perpetrators of violence, on the other hand, believe that the best way to gain respect is to evoke fear and deference; they have low sensitivity and they lack empathy. They frequently hold authoritarian views and rigid rules of “order” with no tolerance for spontaneous or non-fixed rules [11]. Violence is a very personal, sensitive and embarrassing topic, and this could be the reason why the interviewees did not respond to the questions which proved to be particularly personal or sensitive for them. However, it is noteworthy that they decided to participate in the study. This a confirmation that the issues concerning violence are extremely important. 

During the study the respondents were asked about their personal experiences related to the phenomenon of domestic violence. The respondents’ attitude to violence was examined in three dimensions: as victims of domestic violence, as perpetrators of domestic violence and as witnesses to domestic violence. 

Statistically, Polish respondents were more frequently involved in domestic violence as witnesses and victims, which was confirmed by χ^2^ test. There were no significant statistical differences in the role of the perpetrator of violence, although this answer was more often indicated by students from Poland. The statistical calculations in Table 1 did not take into account the lack of respondents’ answers, but scientific reliability requires mentioning that 12.6% of the respondents from Belarus and 16.7% from Poland refused to provide answers concerning their personal experience as witnesses to violence. A total of 11.7% of the Belarusian respondents and 11.2% of the Polish respondents refused to answer the question ‘Have you been a person using violence against your loved ones?’, and 8.7% of the Belarusian respondents and 11.4% of the Polish respondents refused to answer the question ‘Have you been a person experiencing violence from your close family members?’. The refusal to answer the questions can be interpreted in two ways: the respondents, despite anonymity being ensured, did not want to comment on their personal experiences regarding domestic violence, or they were not fully aware of what behaviour qualifies as domestic violence. While expanding this topic, it may be concluded that students who have never been involved in violence (witnesses, victims of violence, perpetrators of violence) find it easier to indicate sanctions imposing widespread and universal access to information about the perpetrators of violence. This could be compared to the Sex Offenders Register which exists in Poland. The Act of 13 May 2016 on combating the threats of sexual crimes defines specific protection measures aimed at counteraction of the threats of sexual crimes. The register consists of three separate databases: (1) Public access—this is a database with unlimited access, which contains information about sex offenders. Using this database does not require registration or logging in. (2) Limited access—access to this database is available to courts, prosecutors’ offices, the police and other authorized services and authorities as part of the proceedings and other statutory tasks. (3) The so-called National Commission Register contains information about individuals whose names were entered by the State Commission for clarifying cases of activities against sexual freedom and indecency towards minors under the age of 15 [12]. The public register (with unlimited access) contains the following personal details: name and surname, maiden name, date and place of birth, citizenship, sex, place of residence, place of committing the crime, judgment and penalty issued and image. It is worth considering whether this register should be extended to include perpetrators of other types of violence than sexual violence. The answer could be affirmative, but only if the crime is confirmed by a final court judgment. The sanction of making the perpetrators’ personal details and image public was less frequently indicated by the students who have been involved in violence. This may be justified by the very mechanism of violence, which is often concealed by victims of violence out of fear for their safety, and also concealed by the perpetrators of violence for fear of criminal sanctions.

Based on the 95% confidence interval (CI), it can be concluded that the largest number of respondents from both countries surveyed who have been involved in violence as witnesses (85.2–94.8) indicated that an adequate punishment for perpetrators of violence is imprisonment (92.2% BY, 88.9% PL). The respondents who had been involved in violence as perpetrators statistically most often pointed to a restraining order to prevent approaching the family as an appropriate form of punishment, with PL 96.3% more often than BY 73.7%. This form of punishment was also most often indicated by victims of domestic violence, with a similar tendency: 96.2% PL and 79.2% BY. Another sanction suggested by victims of violence was eviction from the place of residence (75.0% BY and 90.0% PL). There were no significant differences between the respondents in the indicated penal consequences depending on the form of involvement in violence (witnesses, perpetrators, victims). It was noticed, however, that people who had never experienced violence more often than those who had been involved in violence pointed to the need to apply social sanctions in the form of publicising the perpetrators’ image (35.5% BY and 39.8% PL) or informing perpetrators’ superiors (79.7% BY and 91.6% PL). In this group of respondents, students from Poland showed less tolerance for perpetrators. The response suggesting that perpetrators of violence should not be punished in any way, although indicated, was statistically insignificant (Table 2). 

The majority of respondents from both Poland and Belarus were of the opinion that action should be taken in the event of suspected use of violence or when witnessing violence, even if it means writing an anonymous report. The response: “report to the police” was statistically significant (*p* < 0.001), which indicates that the police service is regarded as the one that stops domestic violence. The Pearson linear correlation coefficient also indicates statistical significance for the answer “give shelter to victims”—most respondents, especially from Poland (95.2%) consider this action to be an appropriate form of assistance to victims of domestic violence. Persuasion or trying to stop the perpetrator from committing violence was insignificant according to respondents from both countries (Table 3). 

The consequences for perpetrators of domestic violence are considered in two fundamental perspectives—legal and social. In the legal perspective, the strongest relationship between the variables is evident in the responses “imprisonment” and “eviction from accommodation”. In the perspective of social consequences, the correlation of variables occurs in the responses “loss of trust of family and friends” and “breakdown of family/relationship”. Lack of statistical significance is shown by the respondents’ answers regarding very subjective and personal consequences for the perpetrator, i.e., remorse (*p* = 0.9). Given the fact that violence means doing harm intentionally to weaker people, this result is not surprising, nor is the insignificance of the lack of any consequences for perpetrators of domestic violence (*p* = 0.1) (Table 4).

## 4. Discussion

Domestic violence is a very complicated legal, psychological and social phenomenon. The harm is caused by the loved ones, and yet the victims repeatedly justify these acts and remain in toxic relationships for years, which is often caused by deep-rooted beliefs [13]. Victims develop a whole set of symptoms over the years, including low self-esteem, guilt, anxiety, depression, suspicion, loss of hope, emotional numbness, feelings of stigmatization, suicidal thoughts and suicide attempts. People who experience violence are frequently convinced that no-one, including themselves, can help them. The feeling of hopelessness affects the assessment of the relationship and leads to the belief that they are completely dependent on the people using violence [14,15,16]. In the light of research to date, in order to stop domestic violence, victims need to break with the established behavioural patterns of attachment, as well as change their behaviour in terms of communication and proper boundary setting [13]. This is confirmed by the results of the above study on a group of respondents from Poland and Belarus, according to whom the consequences for perpetrators of violence should be imprisonment, a restraining order or eviction from the place of residence. In each case, there is separation of the victim of violence from the perpetrator, which, according to the theory of crisis intervention, not only guarantees safety for the victims, but also makes it possible for them to receive appropriate psychological assistance in order to break the cycle of violence and regain self-respect and independence from the perpetrator in further stages of therapeutic work [17]. Unfortunately, the daily practice of social services and the police shows that perpetrators do not always leave the shared place of residence. In such situations, placement in safe shelter should be guaranteed to victims of violence. In most countries of the world, there are shelters for victims of crime or support centres for victims of domestic violence. There is direct access to them thanks to the “open door” principle and 24 h operation [18]. 

Being a medical student, as a criterion for inclusion of respondents in the research, is important due to the nature and specificity of medical professions. Healthcare workers are professions of social trust. Medical staff frequently act as first contact people for victims of violence and, while taking the patients’ history, they have the opportunity to establish the facts of the incident as well as determine injuries. During the provision of medical assistance, healthcare workers raise the victim’s awareness of their rights, and are in a position to report the incident to the competent services for further initiation of proceedings against the offender. The participation of medical students from the selected countries points to the similarities and differences in their approach to the phenomenon of violence, but also draws their attention to a number of aspects which they needed to consider while providing their responses.

While considering the involvement in domestic violence and the severity of consequences for the perpetrators, two factors are of importance: revealing violence and reacting to violence. The criminological theory of broken windows shows that the lack of response to violation of social norms is conducive to an increase in crime and violation of other norms through “contagiousness”, but also through acceptance. The more acceptance, the less severe the consequences. The more witnesses to the incident, the greater the dispersion of responsibility [19]. This explains why, for many years, in the event of suspected violence, there used to be a lack of reaction or ostensible reaction, not only from direct witnesses, but also from the judiciary, health care, social services or even clergy [17]. As a result of educational activities and continuous raising of public awareness of the phenomenon of domestic violence, this aspect has undergone a significant transformation. The growing numbers of cases of domestic violence disclosed by services do not result from more acts of domestic violence being committed, but from an increase in awareness of what behaviours qualify as domestic violence and of the fact that even if domestic violence is only suspected, it requires intervention [9,17,20,21]. This is also confirmed by the results of the above study. The students from both Poland and Belarus are of the opinion that violence must be responded to and reported, even in an anonymous way. The conducted research also showed that there are still people who do not believe in the effectiveness of actions taken by the appropriate services and who do not believe that the perpetrators of violence will bear any consequences. This is closely related to the type and form of violence used, as it can apply to families who, when observed from the outside, do not give rise to suspicion of the drama taking place in them, because the pathology of the perpetrator’s behaviour is manifested only in family relationships. In these circumstances, the detection rate of violence is low, and the difficulty in collecting evidence works in favour of the perpetrators [22]. 

Some of the respondents of the survey indicated that the perpetrators of violence will not have remorse. This response is substantiated in the literature on the subject. The perpetrators of violence, in order to avoid responsibility and punishment for harm caused to their loved ones, try to deny and hide their aggressive behaviour, while efficiently using the mechanisms of power, control and social norms [18,22]. Such perpetrators are considered decent and righteous people, and all suspicions against them become unbelievable. The main “allies” of perpetrators of violence are secret and silence. If the secret is disclosed, the perpetrator tries to undermine the credibility of the victim, and justifies the harm by accusing the victim of lying, provocation, exaggeration, excessive sensitivity or betrayal [18,22,23]. These mechanisms make it easier for people who have never been involved in violence to comment on legal and social consequences for perpetrators than for people who have been involved in domestic violence, regardless of whether they were the perpetrators, victims or witnesses. The most important aspect of domestic violence prevention is to prosecute and punish the perpetrators. The European Court of Human Rights has found that omission by the state or irregularities in this respect violate Art. 3 of the Convention for the Protection of Human Rights and Fundamental Freedoms. The evidence is the case of Valiuliene v. Lithuania (application no. 33243/07). Loreta Valiuliene, who was a victim of violence, brought charges against her partner. The Lithuanian public prosecutor extended the investigation from 2001 to 2005 to find that the prosecution should have been brought by the victim privately. When the applicant lodged a new request to bring a private prosecution, this was refused as the prosecution had become time-barred and the perpetrator remained unpunished. The European Court of Human Rights awarded the victim EUR 5000 compensation [24]. The Lithuanian context indicates that citizens have the right to enforce protection against violence from the state authorities. Lithuania is a democratic country which, similarly to Poland and Belarus, is of Slavic origin. Both Belarus and Lithuania have close borders with Poland and come from a similar cultural background. In view of the above, the case of Veliuliene v. Lithuania was cited, as no such cases were found in the literature of the countries covered by the study.

The Council of Europe Convention on preventing and combating violence against women and domestic violence [25] also indicates that the key elements of counteracting domestic violence are “immediate response, prevention and protection” interpreted as the need to take legal measures or other actions by competent law enforcement authorities for immediate protection of the victims of domestic violence and the inevitable punishment for the perpetrators.

## 5. Conclusions

Students from both Poland and Belarus who have never been involved in domestic violence indicated social consequences as appropriate punishment for the use of violence more frequently than those who have been involved in violence as witnesses, victims or perpetrators. 

Witnesses and victims of violence were not found to be in favour of a more severe punishment or more serious moral and social consequences than perpetrators of violence. 

The largest group of respondents pointed out that the suitable consequences of using violence should be imprisonment, followed by a restraining order and eviction from the place of residence. 

## Figures and Tables

**Table 1:** Involvement in domestic violence (non-responses were not counted).

Have You Been a Witness to Domestic Violence (in Your Own Family or Another)?						
Response	BY		PL		TOTAL	
	n	%	n	%	n	%
Never	139	68.8	92	44.0	231	56.2
95% CI	62.4–75.2		37.3–50.8		51.4–61.0	
Yes—witness to domestic violence	63	31.2	117	56.0	180	43.8
95% CI	24.8–37.6		49.3–62.7		39.0–48.6	
Total	202	87.4	209	83.3	411	85.3
Chi-square 25.7, *p* = 0.001						
**Have you been a person using violence against your loved ones?**						
Never	182	89.2	190	85.2	372	87.1
95% CI	85.0–93.5		80.5–89.9		83.9–90.3	
Yes—perpetrator of violence	22	10.8	33	14.8	55	12.9
95% CI	6.5–15.0		10.1–19.5		9.7–16.1	
Total	204	88.3	223	88.8	427	88.6
Chi-square 1.5, *p* = 0.2						
**Have you been a person experiencing violence from your close family members?**						
Never	181	85.8	161	73.2	342	79.4
95% CI	81.1–90.5		67.3–79.0		75.5–83.2	
Yes—victim of violence	30	14.2	59	26.8	89	20.6
95% CI	9.5–18.9		21.0–32.7		16.8–24.5	
Total	211	91.3	220	87.6	431	89.4
Chi-square10.4, *p* = 0.001						

**Table 2:** The relationship between the form of involvement in domestic violence and the type of sanctions.

People Involved in Domestic Violence at Least Once	Imprisonment		Eviction from Accommodation, (Even If Perpetrator Is the Owner or Co-owner)		Informing the Employer, School or University		Posting Pictures of Perpetrators of Violence on Social Networks, with Information on Violence		Restraining Order		No Punishment	
**Witnesses to domestic violence**	**BY**	**PL**	**BY**	**PL**	**BY**	**PL**	**BY**	**PL**	**BY**	**PL**	**BY**	**PL**
	92.2 (84.8–99.5)	88.9 (82.7–95.1)	69.4 (56.5–82.3)	89.5 (83.3–95.6)	79.6 (68.9–90.4)	89.0 (82.9–95.1)	25.5 (13.5–37.5)	41.8 (31.6–51.9)	76.5 (64.8–88.1)	96.2 (92.6–99.9)	7.6 (0.4–14.7)	0
	90.0 (85.2–94.8)		82.6 (76.5–88.8)		85.7 (82.5–92.9)		35.9 (27.4–43.1)		89.8 (85.1–94.5)		7.6 (0.4–14.7)	
**Perpetrators of domestic violence**	83.3 (66.1–100)	80.8 (65.6–95.6)	77.8 (58.6–97.0)	90.5 (77.9–103.0)	65.0 (44.1–85.9)	84.6 (70.8–98.5)	29.4 (7.8–51.1)	32.0 (13.7–50.3)	73.7 (53.9–93.5)	96.3 (89.2–103.0	11.1 (3.4–25.6)	0
	81.8 (70.4–93.2)		84.6 (73.3–95.9)		76.1 (63.8–88.4)		31.0 (17.0–44.9)		87.0 (77.2–96.7)		11.1 (3.4–25.6)	
**Victims of domestic violence**	91.7 (80.6–100)	80.0 (68.9–91.1)	75.0 (57.7–92.3)	90.0 (81.7–98.3)	88.5 (76.2–100.7)	81.5 (71.1–91.8)	30.4 (11.6–49.2)	30.6 (17.7–43.5)	79.2 (62.9–95.4)	96.2 (91.1–101.4)	4.0 (−3.7–11.7)	0
	83.8 (75.4–92.2)		85.1 (77.0–93.2)		83.8 (75.7–91.8)		30.6 (22.7–46.0)		90.9 (84.5–97.3)		4.0 (−3.7–11.7)	
**People who have never been involved in domestic violence**	86.1 (80.1–92.0)	95.6 (91.9–99.4)	71.3 (63.5–79.1)	94.9 (91.0–98.9)	79.7 (72.9–86.5)	91.6 (86.6–96.6)	35.5 (27.1–43.9)	39.8 (30.8–48.9)	79.0 (71.9–86.2)	99.2 (97.6–100)	7.7 (3.1–12.3)	0
	49.4 (43.1–55.7)		82.6 (77.9–87.3)		85.3 (81.0–89.7)		37.6 (31.4–43.7)		89.1 (85.2–93.0)		7.7 (3.1–12.3)	

**Table 3:** Opinions on action to be taken in the event of suspected violence or when witnessing violence.

Action, % 95% CI	Response	A Group of Respondents from the Country		Pearson Chi-Square
		BY	PL	
**Write an anonymous report**	No	9.6 (5.1–14.0)	10.3 (6.0–14.5)	0.1 (*p* > 0.05)
	Yes	90.4 (86.0–94.9)	175 (89.7%) (85.5–94)	
**No action**	No	94.7 (91.5–97.9)	99.1 (97.9–100.0)	7.2 (*p* < 0.01)
	Yes	5.3 (2.1–8.5)	0.9 (−0.3–2.1)	
**Report to the police**	No	24.7 (11.8–31.6)	4.0 (1.3–6.7)	32.9 (*p* < 0.001)
	Yes	75.3 (68.4–82.2)	96.0 (93.3–98.7)	
**Report to social assistance centre**	No	14.6 (9.4–19.8)	8.0 (4.2–11.8)	4.2 (*p* < 0.05)
	Yes	85.4 (80.2–90.6)	92.0 (88.2–95.8)	
**It is better not to interfere**	No	85.5 (80.2–90.7)	99.1 (97.8–100.3)	27.0 (*p* < 0.001)
	Yes	14.5 (9.3–19.8)	0.9 (−0.4–2.2)	
**Try to stop the perpetrator or explain that they must not use violence**	No	22.6 (16.3–29.0)	21.8 (15.6–28.0)	0.1 (*p* > 0.05)
	Yes	77.4 (71.1–83.7)	78.2 (72.0–84.4)	
**Provide shelter to victims**	No	24.4 (17.6–31.1)	4.8 (1.6–8.0)	25.3 (*p* < 0.001)
	Yes	75.6 (68.9–82.4)	95.2 (92.0–98.5)	

**Table 4:** Consequences for perpetrators of domestic violence in the perceptions of students from Poland and Belarus.

Consequence	Response	Respondents from Country		Pearson Chi-Square
		BY	PL	
**Loss of trust of family and friends**	No	11.9 (7.3–16.4)	1.3 (−0.2–2.7)	21.3 (*p* < 0.001)
	Yes	88.1 (83.6–92.7)	98.7 (97.3–100.2)	
**Imprisonment**	No	23.9 (17.8–30.1)	10.2 (6.3–14.2)	13.9 (*p* < 0.001)
	Yes	76.1 (69.9–82.3)	89.8 (85.8–93.7)	
**Remorse**	No	34.1 (27.1–41.1)	29.4 (23.0–35.8)	0.9 (*p* > 0.1)
	Yes	65.9 (58.9–72.9)	70.6 (64.2–77.0)	
**None**	No	86.0 (80.7–91.3)	85.6 (80.8–90.5)	0.1 (*p* > 0.1)
	Yes	14.0 (8.7–19.3)	14.4 (9.5–19.2)	
**Breakup of family/relationship**	No	15.3 (10.2–20.5)	2.7 (0.6–4.8)	21.3 (*p* < 0.001)
	Yes	160 (84.7%) (79.5–89.8)	217 (97.3%) (95.2–99.4)	
**Eviction from accommodation**	No	27.0 (20.2–33.7)	11.3 (6.9–15.8)	14.4 (*p* < 0.001)
	Yes	73.0 (66.3–79.8)	88.7 (84.2–93.1)	

## Data Availability

Data available on request from the authors.
